# Autumnal leaf senescence in *Miscanthus* × *giganteus* and leaf [N] differ by stand age

**DOI:** 10.1093/jxb/erv129

**Published:** 2015-04-04

**Authors:** Nicholas N. Boersma, Frank G. Dohleman, Fernando E. Miguez, Emily A. Heaton

**Affiliations:** ^1^Department of Agronomy, Iowa State University, Ames, IA 50011, USA; ^2^Monsanto Company, 800 N. Lindbergh Blvd., St Louis, MO 63167, USA

**Keywords:** Chilling, chronosequence, CO_2_ assimilation, *Miscanthus × giganteus*, nitrogen, photosynthesis, survival, translocation.

## Abstract

Poor first winter survival in *Miscanthus* × *giganteus* has been anecdotally attributed to incomplete first autumn senescence, but these assessments never paired first-year with older *M*. × *giganteus* in side-by-side trials to separate the effect of weather from stand age. Here CO_2_ assimilation rate (*A*), photosystem II efficiency (Φ_PSII_), and leaf N concentration ([N]) were used to directly compare senescence in first, second, and third-year stands of *M*. × *giganteus*. Three *M*. × *giganteus* fields were planted with eight plots, one field each in 2009, 2010, and 2011. To quantify autumnal leaf senescence of plants within each stand age, photosynthetic and leaf [N] measurements were made twice weekly from early September until a killing frost. Following chilling events (daily temperature averages below 10 °C), photosynthetic rates in first year plants rebounded to a greater degree than those in second- and third-year plants. By the end of the growing season, first-year *M*. × *giganteus* had *A* and Φ_PSII_ rates up to 4 times greater than third-year *M*. × *giganteus*, while leaf [N] was up to 2.4 times greater. The increased photosynthetic capability and leaf N status in first-year *M*. × *giganteus* suggests that the photosynthetic apparatus was not dismantled before a killing frost, thus potentially limiting nutrient translocation, and may explain why young *M*. × *giganteus* stands do not survive winter when older stands do. Because previous senescence research has primarily focused on annual or woody species, our results suggest that *M*. × *giganteus* may be an interesting herbaceous perennial system to investigate the interactive effects of plant ageing and nutrient status on senescence and may highlight management strategies that could potentially increase winter survival rates in first-year stands.

## Highlight

This chronosequence field experiment found unexpected differences in leaf senescence symptoms between different aged *Miscanthus×giganteus* stands, potentially indicating differential senescence with plant age and nutrient status.

## Introduction

Interest in bioenergy derived from dedicated perennial crops has grown substantially in the past decade. Rising fuel prices, increasing awareness of climate change, and instability in major petroleum-producing nations have all contributed to an increased demand for bioenergy. Among perennial energy crops, *Miscanthus × giganteus* (Greef et Deu.) has been shown to be among the highest yielding options for cool temperate regions such as the Midwestern United States, where it produces nearly three times the amount of dry biomass of switchgrass (*Panicum virgatum* L.), another C_4_ grass commonly used for bioenergy (Heaton *et al.*, 2008; Arundale *et al.*, 2014).

In addition to high yields, *M*. × *giganteus* has many other characteristics which also increase its popularity as a biomass crop (Heaton *et al.*, 2004; Somerville *et al.*, 2010). Among these is C_4_ photosynthesis, which can be up to 40% more efficient than C_3_ photosynthesis (Long, 1999). Somewhat uniquely among C_4_ plants, *M*. *× giganteus* is very efficient at maintaining photosynthesis and high productivity at low temperatures (Beale and Long, 1995; Wang *et al.*, 2008; Long and Spence, 2013). Further, the ‘Illinois’ clone, widely used in US trials, seems to tolerate chilling temperatures even better than closely related genotypes, enabling continued leaf extension in young plants moved from 25 °C to 10 °C (Glowacka *et al.*, 2014*a*).

Despite its cold-tolerant photosynthesis, a major challenge in early European *M.* × *giganteus* trials was winter mortality following the first growing season (Clifton-Brown and Lewandowski, 2000; Christian and Haase, 2001). Perhaps it was not surprising that winter survival was an issue for *M*. × *giganteus*, given the tropical to sub-tropical origins of the genus (Scally *et al.*, 2001). However, because planting costs are a major contributor to the overall production costs of this sterile, clonal crop (Khanna *et al.*, 2008), high winter losses that, in some cases, have approached 100% in temperate climates (Christian and Haase, 2001), are not acceptable and will hinder the acceptance of *M.* × *giganteus* as a bioenergy feedstock.

To minimize winter losses through plant breeding or management, it is important first to understand the cause of winter mortality. It has been speculated that young *M.* × *giganteus* does not go dormant (as indicated by autumnal leaf senescence) early enough in the season to avoid the effects of cold temperatures (Christian and Haase, 2001). Leaf senescence allows nutrients, specifically N, to be redistributed to the rhizome (Heaton *et al.*, 2009; Dohleman *et al.*, 2012); if this process does not occur, or is greatly reduced, rhizomes may have inadequate nutrition to survive the winter and re-sprout in the spring (Beale and Long, 1997). Similar observations have been made in Iowa, USA: first-year stands of *M.* × *giganteus* remain bright green late into the autumn, while second- and third-year stands of *M.* × *giganteus* begin yellowing and senescing (personal observation; see Supplementary Fig. S1 at *JXB* online). This observation was also made in early *M*. × *giganteus* trials (Clifton-Brown and Lewandowski, 2000) and more recently (Robson *et al.*, 2012), however, these previous assessments were made using a subjective greenness index, and did not directly compare first-year *M*. × *giganteus* to older *M*. × *giganteus* within the same growing season.

Given that biomass production increases dramatically during the first few years of growth, it may be that N pools are simply diluted in older, larger stands of *M. × giganteus*, and senescence indicators are really measuring differences in N status. Certainly, limited N can decrease leaf longevity and induce senescence (Wolfe *et al.*, 1988; Xu *et al.*, 2012), but senescence is regulated by more than nutrient status (Thomas, 2013). While it is very difficult to separate the interactive effects of plant ageing from nutrient status in the field, a first step would be to test if plants of different ages begin functioning differently at the end of the growing season. Field performance of first-year *M. × giganteus* is rarely reported in the literature and the objective quantification of *M*. × *giganteus* leaf senescence at a biochemical level has not previously been reported to our knowledge. Indeed, most senescence research has been conducted on annual plants or woody perennials. Very little consideration has been given to the effects of age on senescence in herbaceous perennials, especially grasses, even though the processes of senescence and ageing are critically important to their survival, nutrient use efficiency, and stand longevity.

Senescence is a multifaceted, highly regulated phenomenon, and annual senescence of above-ground tissues is critical to winter survival and biomass quality. Within leaves, the majority of proteins and N is bound in the photosynthetic apparatus. These proteins and N are found within the chloroplasts which are dismantled early in senescence (Feller and Fischer, 1994). Therefore, the timing of senescence determines where mineral nutrients are, and when. If senescence does not occur before a killing frost, the majority of above-ground N will be tied up in those tissues and removed from the system at harvest (Wilson *et al.*, 2013*a*). This leads to mineral contaminants in the material being processed into biofuels and limits the nutrients available to the perennating rhizome (Wilson *et al.*, 2013*b*).

Recently, the topics of senescence, ageing, and death were reviewed at a whole-plant level (Thomas, 2013). It was argued that these distinct processes should be considered separately and each process was discretely defined. Thomas states: ‘Over the course of these extremely extended lifetimes, the cycle of initiation, maturation, senescence, and death of individual structural units will have been recurrent, apparently continuing independently of whatever processes determine ageing and longevity of the plant as a whole.’ Our consideration was confined to autumnal senescence and, specifically, that senescence which precedes leaf death in *M*. × *giganteus*, while ageing continues in the perennial rhizome system. This is an example of death of an individual structural unit (e.g. leaves, stems), but a perennation and ageing of the plant as a whole via the rhizome complex.

Do first-year *M.* × *giganteus* stands senesce differently in the first autumn than in subsequent years? To establish a link between stand age and annual autumnal senescence in *M*. × *giganteus*, a chronosequence field experiment was used to ascertain if first-year *M.* × *giganteus* indeed exhibits delayed autumnal leaf senescence relative to older *M.* × *giganteus*. Seeking a quantitative proxy for autumnal leaf senescence, net CO_2_ assimilation rates (*A*; μmol m^–2^ s^–1^), stomatal conductance (*g*_s_; mol m^–2^ s^–1^), photosystem II (PSII) electron transport efficiency (Φ_PSII_; dimensionless), and total N concentration ([N]; %) were measured in leaves to assess the overall integrity of the photosynthetic apparatus during the transitional period of late summer to autumn (the first killing frost) in cohorts of one, two, and three-year old stands of *M. × giganteus*. In contrast to previous studies which investigated *M*. × *giganteus* senescence, here the parameters above were quantified within the same growing season and environment.

## Materials and methods

### Site description and experimental design

*Miscanthus* × *giganteus* (Illinois clone) plots were established at the Iowa State University Hinds research farm near Ames, IA, USA (42°3’32.04’’N, 93°37’0.25’’W). Three individual fields were planted; one each in May of 2009, 2010, and 2011. Each field consisted of eight plots (*n*=8). Each plot within the field established in 2009 was 10.7 × 10.7 m, with *M*. × *giganteus* plants spaced at 0.8 m between and within rows, in an equal spacing grid. Due to space constraints, fields established in 2010 and 2011 were 6.1 × 6.1 m, but with plant spacing equal to that of the 2009 field. Following common best practice at the time of this experiment, no N fertilizer was applied during the course of this experiment.

### Plant material

*Miscanthus × giganteus* planting stock was obtained from Caveny Farm (Monticello, IL, USA), Speedling Inc. (Sun City, FL, USA), and adjacent fields at the Hinds research farm. All material originated from source plants at the University of Illinois at Urbana-Champaign (Glowacka *et al.*, 2014*b*); see Boersma and Heaton (2014*a*) for a full description of material propagation and planting. Briefly, plants were either propagated conventionally by rhizomes, or by stem propagation. Stem propagation involved cutting single node segments from the lower nodes of existing stems and directly planting them into pots. Once established, stem-propagated plants were transplanted at the same time rhizomes were planted. Alongside the present study, differences between plants grown from stem-propagated plants and rhizomes were examined over the course of three years (2009–2011) at the location described here, as well as at two additional locations, and were found to be functionally equivalent, with no significant major differences in morphology, growth or biomass partitioning (Boersma and Heaton, 2014*a*, *b*). Thus, plants of both propagation backgrounds were used for this experiment.

### Photosynthesis and fluorescence measurements

All measurements were made twice weekly on the youngest, fully expanded (as indicated by ligule presence) leaves in full light. Two randomly selected plants were measured in each plot, beginning in early September until a killing frost (late October).

To minimize diurnal variation, measurements were completed within two hours of solar noon during periods of clear skies. Photosynthetic parameters were measured using a portable open-path gas analyser equipped with infrared CO_2_ and water vapour sensors, a red-blue LED light source, and an integrated leaf chamber fluorometer (LI-6400xt; LI-6400–40, Licor®, Lincoln, NE, USA). The environmental conditions within the leaf cuvette were set to ambient temperature, photosynthetic photon flux density, relative humidity, and CO_2_ concentration ([CO_2_]) at the commencement of measurements each day. Measurements were considered steady-state when displayed *A*, *g*_s_, sample H_2_O concentration ([H_2_O]), and sample [CO_2_] stabilized (*A* slope <1.0, stomatal *g*_s_ slope <0.2, sample [H_2_O] coefficient of variation <0.5, and sample [CO_2_] coefficient of variation <0.5), typically in <5min.

Modulated chlorophyll fluorescence was simultaneously measured with other photosynthetic parameters. Using the steady-state chlorophyll fluorescence and the modulated chlorophyll fluorescence following a saturating pulse, the ratio of absorbed photons used in photochemistry was determined, i.e. Φ_PSII_, following Genty *et al.* (1989). Decreasing Φ_PSII_ is often used to diagnose stress (Maxwell and Johnson, 2000), but it has also been correlated to senescence in rice (Rao *et al.*, 2003), and the maintenance of PSII is consistent with a lack of early leaf senescence since the chloroplast is among the first organelles dismantled during leaf senescence (Feller and Fischer, 1994).

### Leaf [N] measurements

After photosynthetic measurements were recorded, the measured leaf lamina was excised, dried to a constant mass in a forced air furnace at 50 °C, then ground to 1mm with a cyclone sample mill (UDY Corp., Fort Collins, CO). The total [N] of 100–150mg of ground leaf sample was then determined using combustion analysis (LECO® TruSpec CN elemental analyzer, LECO® Corp., St Joseph, MI, USA).

### Statistical analyses

Data were analysed by repeated measures analysis of variance (ANOVA) using PROC MIXED in SAS® version 9.4 (SAS Institute Inc. Cary, NC, USA). Fixed effects of stand age, date of measurement and their interaction were tested by the residual error. Different variance structures were modelled for each response, and the best (as indicated by the lowest Akaike information criterion value (AIC)) model was used to account for the variability associated with non-independence of repeated measures analyses. Least squares means were calculated and used for mean comparisons tests where appropriate. In order to test the hypothesis that *M*. × *giganteus* stands from different ages were under different water-stress environments, a regression analysis was conducted of the relationship between *A* (response variable) and *g*_s_ (independent variable). The relationship, i.e. the slope of the regression (also interpreted as the instantaneous water use efficiency) were tested separately for each year using the lme function in the nlme package in R (Pinheiro and Bates, 2000; Pinheiro *et al.*, 2013).

## Results

**Fig. 1.**
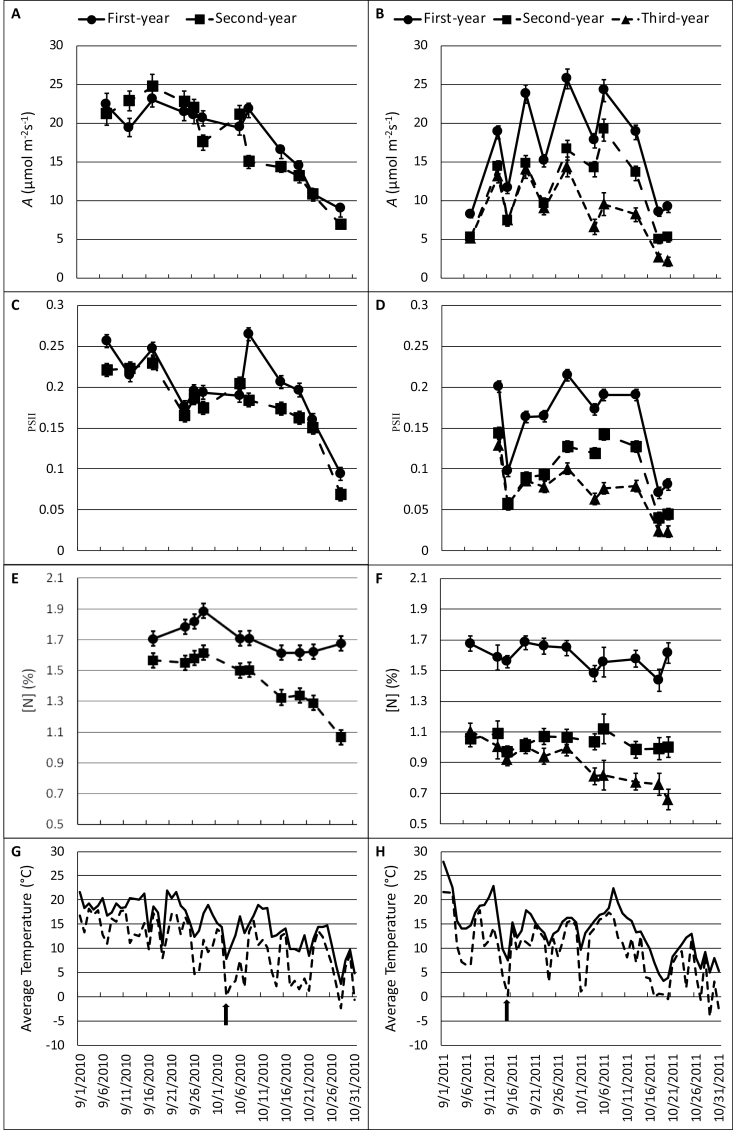
*Miscanthus* × *giganteus* senescence response to date and average daily temperature. Net CO_2_ assimilation rate (*A,* μmol m^–2^ s^–1^) (A, B), photosystem II efficiency (Φ_PSII,_ dimensionless) (C, D), and total leaf N ([N], %) (E, F) were measured in autumn 2010 (A, C, E) and 2011 (B, D, F). Measurements were made on two randomly chosen plants per plot and were averaged within eight plots for first-year (closed circles), second-year (closed squares) and third-year (closed triangles) *M*. × *giganteus* on each date. Points plotted indicate the mean of these eight (*n*=8) observations within each stand age and date combination. Error bars indicate ±1 standard error of the mean. Average daily temperatures (solid line) and daily low temperatures (dotted line) were recorded at an adjacent (6.3 km NE) weather station and acquired from the Iowa Environmental Mesonet (http://mesonet.agron.iastate.edu/). Arrows indicate the first ‘cold-shock’ day of each growing season.

**Fig. 2.**
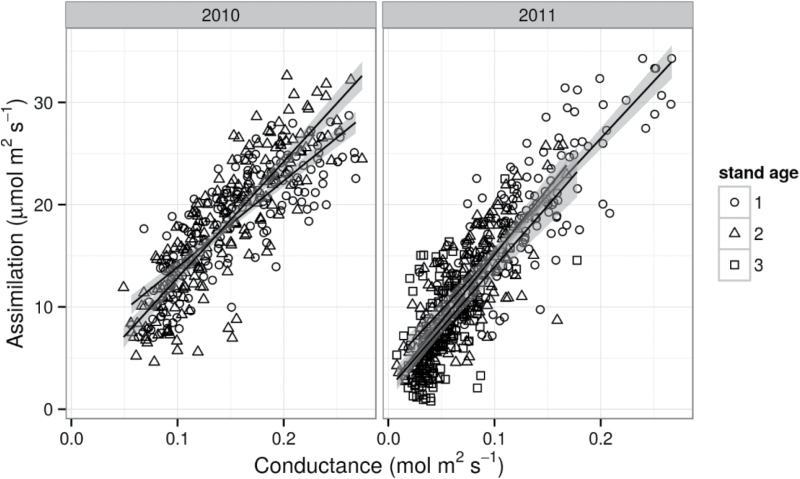
Linear regression analysis of stomatal conductance (*g*_s_, mol m^–2^ s^–1^) and net photosynthetic assimilation rate (*A*, μmol m^–2^ s^–1^) for first-year (circles), second-year (squares), and third-year (triangles) *M*. × *giganteus*. Points plotted are individual measurements taken throughout 2010 (left) and 2011 (right). Lines indicate the linear regression best fit and grey shading indicates a 95% confidence interval of the line.

In this study, first-year *M.* × *giganteus* maintained greater photosynthetic capacity during late autumn than second- and third-year *M*. × *giganteus* as evidenced by a greater rebound in photosynthetic rates and Φ_PSII_ in the days following a ‘cold-shock’ (days with temperature averages of <10 °C; Fig. 1). In addition, second- and third-year *M.* × *giganteus* typically maintained lower photosynthetic rates and leaf [N] throughout autumn until a killing frost. Leaf [N] differences between stand ages were apparent early in the autumn, especially in 2011 when measurements probably commenced too late to capture any divergence between stand ages. Regression analysis showed that the relationship (slopes) of *g*_s_ and *A* was slightly different in first and second-year plants in 2010 (*P* <0.0001), but in 2011 when photosynthetic differences were more pronounced between the different stand ages, there were no differences in the relationship of *g*_s_ and *A* (*P*=0.1600; Fig. 2).

Following ‘cold-shock’ days (indicated by an arrow, Fig. 1G, H), differences in photosynthetic parameters were especially evident, and first-year *M*. × *giganteus* showed *A* and Φ_PSII_ levels comparable to the pre-chilling conditions once temperatures warmed. This rebounding effect happened within a few days after the cold temperatures and did not correspond directly to changes in [N] (Fig. 1). *Miscanthus × giganteus* of different ages also exhibited different levels of *A* (*P* <0.0001), Φ_PSII_ (*P* <0.0001), and leaf [N] (*P* ≤0.0587) as the season progressed from late summer to a killing frost. First-year *M.* × *giganteus* stands typically maintained higher levels of *A* and Φ_PSII_ throughout the autumn (Fig. 2A–D) than did older stands. Differences in these attributes increased between stand ages after the average daily temperature fell below 10 °C (Fig. 2A–D, G, H). For example, on 15 September 2011, the average daily temperature fell to 7.6 °C; the next measurement date was 19 September 2011, which was substantially warmer, 18.9 °C. Net CO_2_ assimilation rates for first-year plants on 19 September 2011 averaged 23.8 μmol m^–2^ s^–1^ but *A* in second- and third-year plants, which did not differ from each other (*P*=0.6611), averaged 41% lower than first-year plants (14.1 μmol m^–2^ s^–1^; *P* <0.0001). The recovery of Φ_PSII_ after cold days in first-year, but not older, *M.* × *giganteus* was especially evident (Fig. 2C, D, G, H). For example, on 3 October 2010, the daily temperature fell to 7.9 °C. When measured five days later, the average temperature had warmed to 15.1 °C. After this warming period, first-year *M.* × *giganteus* Φ_PSII_ rebounded to 0.26, while second-year *M.* × *giganteus* maintained a 31% lower Φ_PSII_ (0.18; *P* <0.0001).

Leaf [N] remained higher in first-year stands than in second- and third-year stands, and decreased very little throughout the growing season (Fig. 2E, F). For example, in 2010, first-year stands began and ended the autumn with 1.7% leaf [N]. However, although second-year *M.* × *giganteus* began the same season with a similar leaf [N] to first-year *M.* × *giganteus* (1.6%; *P*=0.5613), it significantly decreased by the end of the season to 1.1% (*P* <0.0001). In 2011, differences in leaf [N] between first-year and second- and third-year *M.* × *giganteus* were already present at the first date of sampling (*P* <0.0001), and continued to diverge. By the end of the season, third-year *M.* × *giganteus* leaves had significantly lower [N] than second-year *M.* × *giganteus* (*P*=0.0004), and second-year *M.* × *giganteus* leaves had a significantly lower [N] than first-year *M.* × *giganteus* (*P* <0.0001).

## Discussion

Leaf photosynthetic performance and [N] were used here as quantitative proxies for whole-plant autumnal senescence in a chronosequence of clonally propagated *M.* × *giganteus*. Although previous research has alluded to differential timing of senescence as *M*. × *giganteus* ages, to our knowledge, these data represent the first direct and quantitative field comparisons of senescence symptoms in first-year *M.* × *giganteus* to older stands within the same growing season and environment.

Prolonged differences in photosynthetic performance of different aged plants may be attributed to leaf [N] differences that were maintained throughout the season, probably due to an overall dilution of N in the older, larger plants. However, N status does not seem to explain short-term differences in photosynthetic performance, especially following the coldest temperatures experienced to date within a growing season. These ‘cold-shock’ responses are consistent with the results of controlled environment studies which showed that newly planted *M.* × *giganteus* exhibited increased levels of pyruvate phosphate dikinase (PPDK) a few days after transfer from 25 °C to 14 °C (Wang *et al.*, 2008). Wang *et al.* (2008) showed that increased PPDK in these plants corresponded to an ability to maintain *A* at 80% of the rate they had prior to moving to cold temperatures, even while continually growing at 14 °C. Further, when similar young plants were grown at 14 °C but measured at 25 °C, they had virtually identical *A* rates as those grown at 25 °C (Naidu *et al.*, 2003). Although our experiment was conducted in the field, similar responses were found in *A*. After the coldest days (<10 °C), first-year stands of *M.* × *giganteus* had higher rates of *A* even while temperatures remained cool and especially when temperatures warmed. By contrast, older stands maintained lower *A* while temperatures remained cool and showed relatively modest increases in *A* following a warming period. Perhaps increased PPDK, which allows first-year *M.* × *giganteus* to maintain high *A*, may not be as pronounced in second- and third-year field-grown *M.* × *giganteus*.

This hypothesis is also consistent with the finding that, over a short time period (a few days from ‘cold-shock’ to rebounding effect), photosynthetic parameters changed quickly in first-year plants, but over that same time period there was no change in [N] in either first-year or older plants. Perhaps in younger *M*. × *giganteus*, the intact photosynthetic apparatus continued functioning while, in older plants, the photosynthetic apparatus is either dismantled, or the [N] is too low to increase PPDK and maintain photosynthesis following ‘cold-shocks’.

In addition to the rapid rebounding of first-year plants, it was found that first-year *M.* × *giganteus* maintained higher levels of *A*, Φ_PSII_, and leaf [N] throughout the autumn, while levels of these parameters declined in older stands. Our hypothesis is that senescence in first-year *M.* × *giganteus* is delayed or absent, but *M. × giganteus* gains senescence competence as it ages. These findings are consistent with the subjective greenness indices reported by Robson *et al.* (2012), which showed high greenness ratings until a killing frost for younger *M.* × *giganteus* (and other *Miscanthus* species), but lower greenness ratings as plants aged, and bolster anecdotal remarks made by Beale and Long (1995) that senescence in the second season was more apparent than in the first season. However, neither of these trials compared different aged stands side-by-side.

Reduced senescence in first-year *M.* × *giganteus* is further supported by our finding that, as *M.* × *giganteus* ages, leaf [N] decreased more rapidly in the autumn, consistent with the breakdown of photosynthetic proteins and translocation to the perennating rhizome (Beale and Long, 1997). Although a decreasing pattern was not always observed in older plants, e.g. second-year *M.* × *giganteus* in 2011, the leaf [N] values found here are consistent with the N translocation timing of established *M.* × *giganteus* shown by others (Beale and Long, 1997; Heaton *et al.*, 2009; Dohleman *et al.*, 2012), and suggests N translocation from above-ground biomass may be mostly complete by August. Interestingly, the 1.6–1.7% leaf [N] observed here for first-year plants, just prior to a killing frost, was very similar to the leaf [N] found in June for established *M.* × *giganteus* in previous trials (~1.5%) (Dohleman *et al.*, 2009, 2012; Heaton *et al.*, 2009). Likewise, the leaf [N] of 0.8–1.0 % observed here for established *M.* × *giganteus* during late October was consistent with Dohleman *et al.* (2012), who found a leaf [N] of ~0.7% during October for established *M.* × *giganteus*.

Many factors contribute to autumnal leaf senescence, including stress from limited water and/or N availability. Although *g*_s_ did appear to be slightly greater in first-year plants in 2010, in 2011, when photosynthetic differences were more pronounced between the different stand ages, there were no differences in the slopes of *A* to *g*_s_, indicating that stand ages did not respond differently to water stress during this period of low evaporative demand (Fig. 2). It is likely that N dilution in older, larger stands contributed to the leaf [N] observed here. These fields received no supplemental N fertilization, and fertility management may be a way to influence senescence in *M*. × *giganteus*.

Our results suggest that leaf senescence of first-year *M.* × *giganteus* is significantly delayed or reduced compared with older *M.* × *giganteus*. Delayed senescence may have positive and negative effects on *M*. × *giganteus* production in its first season. It allows the crop to maximize the growing season by maintaining photosynthetic tissue, however, in the absence of translocation, above-ground nutrients may be lost and increase the potential for overwintering mortality. Given the propensity of *M. × giganteus* stands to have high winter mortality rates after the first season, it is hypothesized that increasing senescence and nutrient translocation to the rhizome system would be more beneficial to *M*. × g*iganteus* production.

Although our senescence results are consistent with what others have anecdotally noted for *M*. × *giganteus*, they seem to contradict Thomas (2013) who states: ‘Over the course of these extremely extended lifetimes, the cycle of initiation, maturation, senescence, and death of individual structural units will have been recurrent, apparently continuing independently of whatever processes determine ageing and longevity of the plant as a whole.’ We found that senescence of individual structural units (leaves) does, in fact, appear to be dependent on the ageing of the plant as a whole. Given that perennial C_4_ grasses like *M. × giganteus* are increasingly important as bioenergy crops, more study is warranted to separate the interactive effects of plant ageing from nutrient status in the field. It may be possible that fertilization regimes could be used to manage not only crop productivity and quality, but also senescence and crop survival.

## Supplementary data

Supplementary data can be found at *JXB* online.

Supplementary Fig. S1. First (left) and second-year (right) *M.* × *giganteus* stands.

Supplementary Data
