# Supplementary material for: Autumnal leaf senescence in Miscanthus × giganteus and leaf [N] differ by stand age
Source: J Exp Bot. 2015 Apr 4;66(14):4395–401. doi: 10.1093/jxb/erv129 (PMC4493784; doi:10.1093/jxb/erv129)
Supplement: Supplementary Data [file supp_66_14_4395__index.html]

Autumnal leaf senescence in Miscanthus × giganteus and leaf [N] differ by stand age — Autumnal leaf senescence in Miscanthus × giganteus and leaf [N] differ by stand age — Supplementary Data 

# Autumnal leaf senescence in *Miscanthus* × *giganteus* and leaf [N] differ by stand age

## Supplementary Data

Data files

**Files in this Data Supplement:**

- Supplementary Data - Supplementary Data
