# Supplementary material for: Autumnal leaf senescence in Miscanthus × giganteus and leaf [N] differ by stand age
Source: J Exp Bot. 2015 Apr 4;66(14):4395–401. doi: 10.1093/jxb/erv129 (PMC4493784; doi:10.1093/jxb/erv129)
Supplement: Supplementary Data [file supp_erv129_jexbot138578_file001.pdf]

Title: Autumnal leaf senescence in Miscanthus × giganteus and leaf [N] differ by stand age

Authors: **Nicholas N Boersma, Frank G Dohleman, Fernando Miguez, and Emily Heaton**

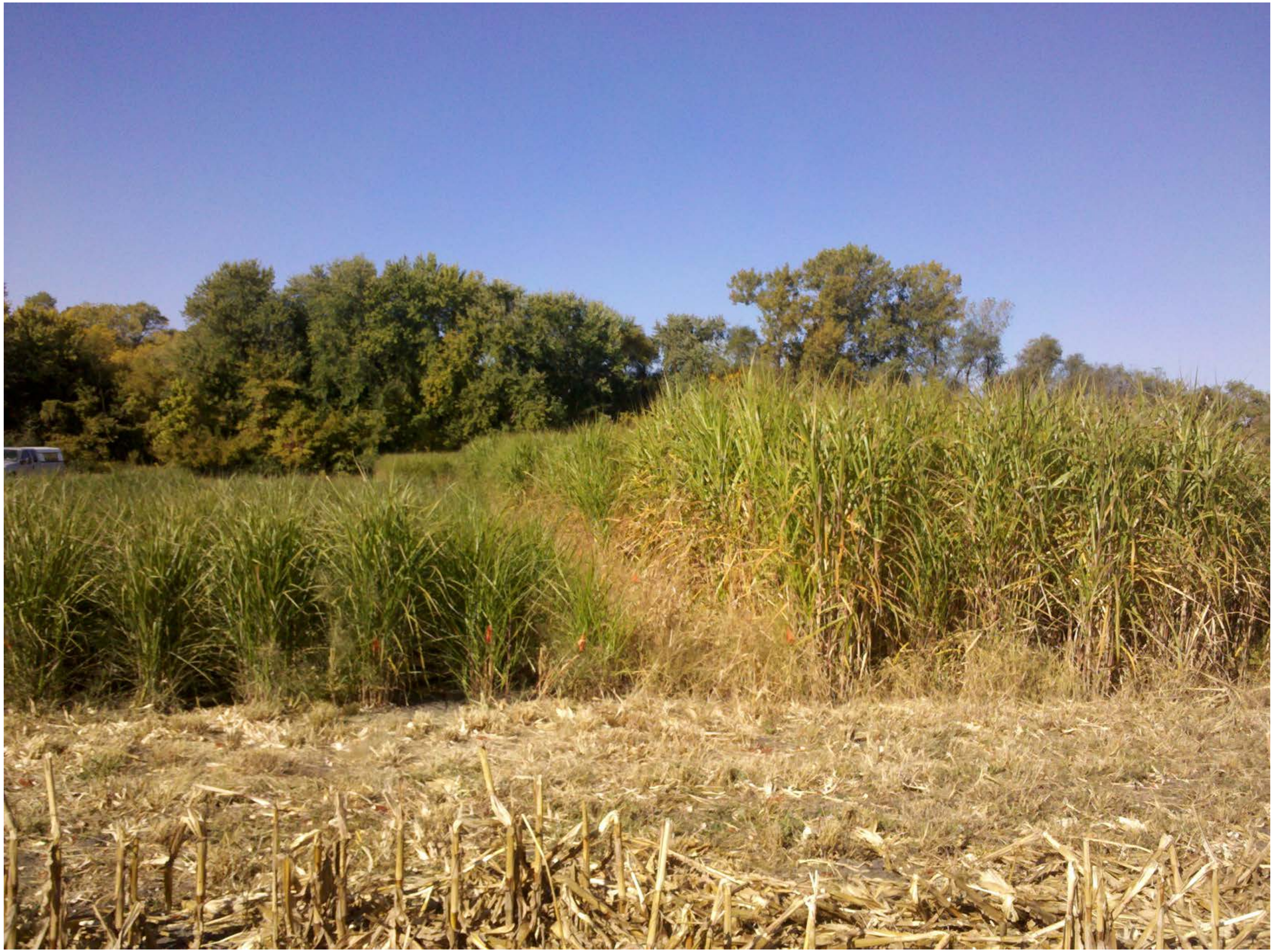

Supplementary Fig. S1. First (left) and second-year (right) *M.* × *giganteus* stands. First-year *M.* × *giganteus* remains green long into the autumn, whereas second-year *M.* × *giganteus* begins yellowing and senescing which is especially evident near the bottom of the plants. Note corn (*Zea mays* L.) has been harvested prior to this photo. Photo credit: Nicholas Boersma, 4 October 2011.
